# Autophagy-mediated tumor cell survival and progression of breast cancer metastasis to the brain

**DOI:** 10.7150/jca.50137

**Published:** 2021-01-01

**Authors:** Aparna Maiti, Nitai C. Hait

**Affiliations:** Division of Breast Surgery and Department of Surgical Oncology, Department of Molecular & Cellular Biology, Roswell Park Comprehensive Cancer Center, Buffalo, New York, 14263, USA.

**Keywords:** breast cancer, brain metastases, astrocytes, autophagy, cell survival

## Abstract

Brain metastases represent a substantial amount of morbidity and mortality in breast cancer (BC). Metastatic breast tumor cells committed to brain metastases are unique because they escape immune surveillance, can penetrate the blood-brain barrier, and also adapt to the brain tissue microenvironment (TME) for colonization and outgrowth. In addition, dynamic intracellular interactions between metastatic cancer cells and neighboring astrocytes in the brain are thought to play essential roles in brain tumor progression. A better understanding of the above mechanisms will lead to developing more effective therapies for brain metastases. Growing literature suggests autophagy, a conserved lysosomal degradation pathway involved in cellular homeostasis under stressful conditions, plays essential roles in breast tumor metastatic transformation and brain metastases. Cancer cells must adapt under various microenvironmental stresses, such as hypoxia, and nutrient (glucose) deprivation, in order to survive and progress. Clinical studies reveal that tumoral expression of autophagy-related proteins is higher in brain metastasis compared to primary breast tumors. In this review, we outline the molecular mechanisms underlying autophagy-mediated BC cell survival and metastasis to the brain.

## Introduction

Metastasis is a complex interlinked multi-step process and accounts for 90% of BC mortality 1, 2. Breast cancer brain metastasis (BCBM) occurs in approximately 15-30% of women with late-stage BC, and it is often seen in women with more aggressive cancers such as triple-negative or HER2+ 3.

Available therapies for brain metastasis include whole-brain radiation therapy (WBRT) and stereotactic radiosurgery (SRS) 4. Although SRS offers the additional ability to treat tumors in more of a normal tissue sparing manner compared to WBRT, however, combining the two techniques have been shown to improve survival and local tumor control in select patients. Current clinical management of brain metastases has limitations in controlling the disease and can impair neurocognitive function, with patient's survival less than 12 months after diagnosis 5. Thus, this underscores the need to understand this brain-specific metastatic process to better identify women at risk of developing brain metastasis, and finding ways their occurrence altogether. Therefore, an urgent need exists to identify new genetic signatures and underlying mechanisms of BCBM and to discover novel molecular targets to prevent this process.

In BC, it may take years or decades for cancer cells to precede distant relapse 6, 7, suggesting BC cells initially lack the full competence for outgrowth in distant organs until they slowly they get modified to survive in secondary sites. Metastatic tumor cells must first overcome the blood-brain barrier (BBB), which acts as a selective interface between the peripheral circulation and the central nervous system 8, 9. Metastatic tumor cells must adapt themselves to a different microenvironment from that of the primary site 10, 11. How breast tumor metastatic cells adapt a specific niche in the brain for their self-renewal is an intriguing question for therapeutic and prognostic purposes. Although this complex topic requires further investigations, several pieces of evidence report on the role of autophagy in different stages of breast tumor metastasis 12-14.

In this review, we discuss that autophagy may be the mechanism responsible for metastatic cancer cells undergoing adaptation to escape all adverse environments. With the help of astrocytes, metastatic cancer cells colonize in the brain.

## Autophagy helps cancer cells to be resistant to stressors

Macroautophagy (autophagy) is a well-established conserved mechanism that targets intracellular components for degradation and recycles protein and organelles to generate amino acids, nucleotides, fatty acids, sugars, and ATP to survive under various stresses, such as hypoxia and nutrient (glucose) deprivation 15, 16. Growing literature suggests autophagy plays a complex role in tumorigenesis 17. Autophagy has been implicated and plays a crucial role in preserving physiological tissue homeostasis by multiple mechanisms, including enhancing metabolic/redox homeostasis, and maintaining stemness by preventing senescence, with these effects suppressing malignant transformation 18-20. Most early disseminated cancer cells are detected in the bone marrow of BC patients and have a putative BC stem cell phenotype 21. Autophagy is shown to act as both pro-metastatic and anti-metastatic factors 13, 22-24. In the early stages of tumor metastasis, autophagy has been shown to be linked with anti-metastasis via the limitation of cancer necrosis and inflammatory responses 25, 26.

However, recent studies have suggested that autophagy promotes multiple steps in tumor progression, survival, and colonization in distal organs 13, 22, 27, 28. Autophagy is activated in the central part of solid tumors, where cells are exposed to extremely stressful conditions, including hypoxia and nutrient deprivation. Autophagy helps cancer cells to overcome these stresses 29-31. During nutrient deprivation, autophagy is enhanced and acts to promote tumor survival and growth in advanced cancers 32, 33. Autophagy manages the high metabolic demands of proliferating tumors by recycling intracellular components to supply metabolic substrates 34, 35. Therefore, autophagy contributes to tumor-cell survival by enhancing the nutrient supply to meet metabolic demands affording them stress tolerance. Oxidative stress responses involving PKR-like endoplasmic reticulum kinase (PERK)-autophagy survival signaling has been shown to contribute to mammary gland development and tumorigenesis (reviewed in 36). Hyperactivation of the mammalian target of rapamycin complex 1 (mTORC1) promotes BC progression through enhancing glucose starvation-induced autophagy and AKT signaling 37. One of the crucial regulators of autophagy is mTORC1, linked with tumor progression, metastasis, and metabolism 38. Published results have implicated mitochondrial complex I activity modulates AKT/mTORC1-mediated autophagy and BC progression and metastasis to the distant organs, including the brain 39.

Other published papers show that autophagy markers are easily detected in ductal carcinoma *in situ* lesions from human patients 40, 41. Further observations suggest that autophagy regulation and biology in BC could be more complicated. Published results have also implicated defective autophagy in breast tumors could also enhance tumorigenesis 18, 42, 43. On the other hand, the inhibition of autophagy or knockdown of autophagy genes can also result in tumor-cell death 44, suggesting a very promising target in BC treatment 43. Several studies have revealed that anticancer drug resistance increases via the upregulation of autophagy, which functions as a protective mechanism in cancer cells undergoing anticancer therapy 45, 46. Inflammation is also induced by autophagy in the cancer microenvironment and cancer adjacent cells, leading to cancer progression. Inflammatory regulators are increased in the tumor microenvironment, which contributes to tumorigenesis 47. Some studies show that inflammation triggers high levels of reactive oxygen species (ROS) in cancer cells, macrophages, and other immune cells. These cells are in terms of secrete chemokines and cytokines, including interleukin-6 (IL-6), tumor necrosis factor-α (TNFα), interleukin-10 (IL-10), and transforming growth factor-β (TGFβ) into the tumor microenvironment (TME) 48. These cytokines play crucial roles in maintaining chronic inflammation, which contributes to cancer progression via inflammation 49. The multifaceted roles of autophagy have been implicated in modulating breast tumor cell motility and invasion 50-54, cancer stem cell viability and differentiation 41, 55, 56, tumor cell dormancy 20, and escape from immune surveillance 57, with emerging functions in establishing the pre-metastatic niche (reviewed in 13). One recent study suggested disseminated dormant BC cells survive and metastasize to distant organs via the autophagy process. Furthermore, results have implicated that inhibition of autophagic flux in the dormant BC cells enhances the accumulation of damaged mitochondria and ROS, which results in programmed cell death 58. Earlier studies using the mammosphere formation by BC cells have suggested that the autophagy-related gene ATG4A promotes a BC stem-like phenotype 59. Autophagy proteins LC3A and LC3B expressions are a common feature of solid tumors and are associated with proliferation, metastasis, and poor outcome 54, 60. The presence of LC3B puncta and HMGB1 gene expression in the malignant cells is found to be correlated with immune infiltration associated with BC progression and predicted residual risk of relapse after adjuvant chemotherapy 57, 61.

Another study suggests that induction of autophagy genes, ATG7 and conversion of autophagy marker (LC3A to LC3B), and tetraspanin proteins (CD9, CD63, CD81), are useful biomarkers and provide novel therapeutic targets for the treatment and prevention of cancer 62. In agreement, one recent study suggested upregulated expression of ATG7 in BC tissue is significantly associated with reduced overall survival (OS), relapse-free survival (RFS), and distant metastasis-free survival (DMFS) in BC patients 63. Another recent study was performed to assess the significance of the expression of autophagy markers, including beclin-1 (BECN1), LC3A, LC3B, and p62 in the molecular subtypes of TNBC patient samples using tissue microarray analysis. Data suggested that among the subtypes of TNBC, the molecular apocrine TNBC subtype showed a higher expression of nuclear p62 and beclin-1 than other markers, which indicated higher autophagy activity 64. Further follow-up studies by another group have suggested that autophagy-related markers are overexpressed in the TNBC patient samples using immunohistochemical analysis 65. In agreement, these pieces of evidence suggest LC3B may play a critical function in the progression of TNBC and could be a useful marker in prognostic evaluation for patients with TNBC 66. Gene expression omnibus database (http://www.ncbi.nlm.nih.gov/geo/) analysis has identified a set of eight autophagy-related genes (BCL2, BIRC5, EIF4EBP1, ERO1L, FOS, GAPDH, ITPR1 and VEGFA) associated with a prognostic signature of BC patients 67. One recent study suggested that LC3B and ATG17 elevated expression in brain metastases are correlated with significantly shorter survival time in TNBC patients 68. These results are in line with a strategy to target autophagy in the treatment of brain metastases in BC patients. Some essential genes and gene products participating in the process of autophagy and BCBM, and their functions, are summarized in Table 1. Collectively, these observations suggest that the autophagic signaling network is involved in BC cell survival under stressful conditions and tumor metastasis.

## Crossing the blood-brain barrier

The metastatic cascade can be divided into two major phases (i) physical translocation of a cancer cell from the primary tumor to the microenvironment of a distant tissue and (ii) colonization. During physical translocation, cancer cells acquire invasive phenotypes, invade the surrounding matrix, and entering the lymph or blood circulation system 1. The bone marrow of patients with BC correlates with increased metastatic burden, aggressiveness, and reduced survival 86. Animal studies have shown that only a tiny percentage of tumor cells are capable of completing the intricate steps of metastasis, the most limiting of which is the colonization of tumor cells at distant sites 87. Autophagy is also a pro-metastatic cancer cell survival factor 13. In order to undergo metastasis, cancer cells must be able to survive and proliferate in the circulatory system and disseminate to secondary sites as well. Therefore, a subset of invasive circulating tumor cells (CTC) that survive the circulation is capable of extravasation through the BBB for metastatic colonization in the brain 88, 89. Cancer cell death undergoes apoptosis due to loss of extracellular matrix (ECM) attachment, called anoikis. Substantial evidence indicates that autophagy enables ECM-detached cancer cells to avoid anoikis and survive 90.

The ability of tumor cells to initiate growth in the brain is dependent mostly on cross-talk between tumor and brain resident cells. A clear understanding of the biological mechanism of the brain microenvironment and how cancer cells change themselves to survive in that environment is critical to tackling the clinical challenges of brain metastasis. As metastatic cancer cells arrive in the brain's vasculature, these cells are challenged to cross the BBB that separates the brain from the general circulation 91. The BBB is surrounded by a thick basement membrane supported by pericytes, which are fibroblast-like cells that are wrapped around endothelial cells connected by tight junctions. Finally, astrocytes form the outer layer of the BBB 92. The BBB's tight junctions are composed of a variety of proteins, including claudins and occludins 93, and possess high electrical resistance 94. The primary function of this barrier is the maintenance of the homeostatic environment for neuronal function 93.

It has been suggested that cancer cell-derived marker proteins are the prognostic factors for organotropic metastasis to the brain 95. In agreement with this concept, recent studies have suggested that brain-specific gene expressions, such as SEMA4D, and mechanisms in the BC CTCs can transmigrate through BBB 84. Other studies have identified genes from BC clinical samples, such as cyclooxygenase COX2 (also known as PTGS2), the epidermal growth factor receptor (EGFR) ligand HBEGF, and the brain-specific 2,6-sialyltransferase ST6GALNAC5 as mediators of BC cell passage through the BBB 83. Cancer cells need to secrete matrix metalloproteinases (MMPs) and other proteases to disrupt the basement membrane and tight junctions as occurs in metastasis to other distant sites 96-98. It has been demonstrated that secretion of MMP2, MMP3, and MMP9 by metastatic BC cells is playing a crucial role in BCBM 99. Cancer cells and tumor-associated cells, such as macrophages, produce a variety of mediators, which facilitate endothelial cell junction openings 100. It has been shown that secreted vascular endothelial growth factor (VEGF) and TGF-β induce opening the endothelial junctions by disrupting the VE‑cadherin-β‑catenin complex 101-103.

A variety of cytokines, chemokines can also destabilize these junctions and inflammatory mediators frequently expressed by cancer cells and tumor-associated cells, including interleukin-1β (IL-1β), TNF-α, interferon-γ (IFN-γ), CCL2, and CXCL8 (reviewed in 104). The interaction between surviving cancer cells and the brain microenvironment plays a crucial role in cancer cell progression to the brain (reviewed in 105). Cancer-derived extracellular vesicles (EVs), including exosomes, are known to mediate cell-cell communications via delivery of their contents, including proteins, mRNAs, and microRNAs (miRNAs) 106, and are reported capable of breaking down the BBB through modulating actin dynamics and promote brain metastasis 107. One such important recent study suggested that brain metastasis of BC cells or lung tumor cell-derived exosomes enriched with a Wnt-related protein, CEMIP, are shown to promote brain metastasis. Exosomal CEMIP upregulated pro-inflammatory mediators such as TNF, PTGS2, and CCL/CXCL in microglia, which promote BBB dysfunction and brain metastasis 108. As indicated 95, 109, brain metastasis is a complex process that is only recently beginning to be understood, that may require activation of multiple pathways to cross the BBB and adapt to the brain environment, that has potential metastatic cancer cells crossing the BBB through mediator signaling and cell-cell interaction in the brain microenvironment to survive and colonize the brain.

## Astrocytes cross-talk with the cancer cells to pass the barrier

Host cells in the microenvironment influence metastasis of tumor cells. Breast tumor metastasis represents a complex interaction between tumor cells and brain endothelial cells, pericytes, microglia, M2 macrophages, and astrocytes. These different cells are capable of tipping tumor biology toward cell extravasation and influencing the biologic behavior of metastatic cell growth and progression by exhibiting and favoring elevated autophagy. Brain metastasis of BC cells is highly adaptable in the brain by expressing brain-specific gene products and mediators that mediate survival in the brain microenvironment 83. There is an urgent need to identify brain-specific new targets for developing novel therapeutics to prevent BCBM. The tumor microenvironment in the brain is represented predominantly by normal astrocytes in the brain tissues 110, 111. Astrocytes are one of the subtypes of glial cells that become activated in response to central nervous system (CNS) stresses. In the brain environment, the homeostatic function of astrocytes includes supporting the BBB 111, 112, delivering nutrients to neurons 113, regulating the brain response to inflammation 114, and protecting neurons from hypoxia 115.

Astrocytes express glucose transporters for allowing glucose to enter into the brain and glycoproteins for pumping drugs and other compounds out of the brain. They also possess the capability to modify the tightness of the BBB in response to stresses like hypoxia 116. After escaping elimination by astrocytes, cancer cells can take advantage of the protective benefits that astrocytes usually support neurons by a variety of mechanisms. Brain metastatic cancer cells can activate the pro-growth and survival effects of astrocytes by secretion of IL-1β 117 or CCL2 118, and have been shown to enhance the differentiation of neural progenitor cells into astrocytes 119. Reactive astrocytes have altered gene expression patterns, including the up-regulation of Glial Fibrillary Acidic Protein (GFAP), which have been frequently observed in metastatic brain tumors in different animal models and human patients (8, 120, and reviewed in 110, 121. The reciprocal cross-talk between brain metastatic cancer cells and astrocytes promote outgrowth and stimulate the therapeutic resistance of cancer cells in the brain 122. The report by Kim et al. 123 showed the first evidence on the role of the interaction of BC cells with astrocytes in promoting brain metastatic transformation (through the up-regulation of different pro-survival genes, including those stimulating autophagy). These cell-cell interactions and cancer cell survival are associated with increased resistance to various chemotherapeutics.

A better understanding of therapeutic resistance and brain metastasis lies in the assimilated function of astrocytes. Numerous studies have demonstrated that a variety of factors produced and secreted by reactive astrocytes can support metastatic cancer cell growth and progression in the brain. These include neurotrophic factors such as TGF-α, ATP, endothelin-1 (ET-1), glutamate, IL-1β, IL-6, TNF-α, macrophage inflammatory protein MIP-2, CCL2, CXCL12, glial cell-derived neurotrophic factor (GDNF), NO, and bioactive lysophospholipid sphingosine-1-phosphate (S1P) 80, 112, 124, and reviewed in 110, 125. The lipid mediator S1P is produced inside cells by the sphingosine kinases (SphK1 and SphK2) and involved in inflammation and cancer progression 126, 127. However, S1P has complex roles in the tumor microenvironment. The brain has the highest levels of S1P, which is predominantly produced by the sphingosine kinase 2 (SphK2) 128, 129. It has been shown cerebellar astrocytes produce S1P in response to bFGF and induces proliferation of astrocytes via extracellular signaling through G-protein coupled receptors (S1PRs) 130.

One recent study suggests that extracellular S1P signaling through S1P receptor 3 (S1PR3) mediates its effect through astrocytic secretion of IL-6 and CCL2, and relaxes endothelial cell adhesion. Astrocytic neuroinflammatory signaling through S1PR3 has been documented to modulate BBB permeability that enhances BCBM 109, 131. This study is important because it suggests that abnormal angiogenesis-related signaling through S1PR3 in cancer cells or astrocytes could impact the permeability of the BBB and provides a potential therapeutic target of BCBM. Another study demonstrated that astrocytes promote brain metastasis of BC by upregulating autophagy signaling pathways via the CXCL12-MIR345-KISS1 axis 80, 132. BCBM progression exhibits low levels of KISS1 expression. Recently published data suggests that KISS1 expression downregulated ATG5/7-mediated survival autophagy and BCBM 80. One published study suggested that KISS1 expression is significantly higher in primary BC compared with brain metastases 81. Together, these studies have suggested that KISS1-mediated survival autophagy is a potential diagnostic marker for BCBM, as well as a new anticancer therapeutics. Cancer cell and astrocyte gap junction communications have shown influencing barrier permeability associated with promoting cancer cell metastasis to the brain 133. Cancer cells express protocadherin 7 (PCDH7), promote carcinoma-astrocyte gap junctions, which then induces IFNα and TNF production and activation of STAT1 and NF-κB pathways in cancer cells to support brain metastasis 133.

## Metabolic adaptation of cancer cells to the brain microenvironment

Metastatic BC cells must overcome the metabolic adaptation challenges to survive and colonize in the brain microenvironment. It has been proposed that tumor cells acquire a metabolic signature adapted for survival at particular metastatic sites, which dictate where they are able to form distal colonies. This hypothesis is consistent with the growing body of evidence showing that the more metabolically flexible the primary tumor cells are, the more likely they are to survive the metastatic process and thrive in distant organs. The mammalian brain utilizes glucose as the primary energy source for its physiological functions, where astrocytes consume glucose, and neurons preferentially take up and oxidize lactate over glucose-derived pyruvate 134. It has been postulated that primary breast tumors that undergo the Warburg effect 135 of metabolic reprogramming become suited to growth in the brain microenvironment 136. Brain metastatic cancer cells exhibit altered gluconeogenesis and oxidation of glutamine/branched-chain amino acids as alternative energy sources for their adaptation in the foreign low-glucose environment 137, 138. They possess an enhanced ability to utilize glutamine as an energy source and as a precursor to cellular building blocks, including purines, pyrimidines, and non-essential amino acids 139, 140. However, it is unknown whether metastatic brain cells possess an intrinsic ability to utilize glutamine better or is a modified phenotype for the adaptation to the brain environment. A mechanistic understanding of the regulation and cause of this increased glutamine utilization can serve as a therapeutic target for BCBM. One recent study discovered glucose-regulated protein GRP94 over-expression is critical for BCBM, which relives metabolic stress in low-glucose conditions by coordinating the unfolded protein response and pro-survival autophagy 82. These studies suggest autophagy-mediated mechanisms could be a potential therapeutic target in BCBM for designing new therapies.

## Targeting autophagy in BC metastasis

Autophagy and regulatory machinery signaling in cancer are explained in many recent reviews 12, 13, 141. As mentioned earlier, the dual roles of autophagy have been documented in BC as well. The loss of autophagy molecule BECN1 is a frequent event in many human tumors, including breast 69, and is associated with the promotion of tumorigenesis 142. Conversely, overexpression of BECN1 in the MCF-7 BC cell line decreases tumorigenesis 42. In contrast, autophagy has been shown to enhance breast tumor progression and metastasis. For example, published results suggest that mammary epithelial conditional knockout of the essential autophagy protein FIP200 has reduced tumorigenesis and metastasis in the MMTV-PyMT mouse model 44, 143. Another study suggests that DNA damage and oxidative stress-mediated BC cell survival occur through autophagy and the involvement of p53 144. Recent studies suggest that the effector of the Hippo signaling pathway yes-associated protein (YAP) is involved in promoting autophagy-mediated TNBC cell survival and metastatic properties 145, 146. Altering autophagic flux 141 by manipulating autophagy signaling has been recognized as an attractive cancer therapy strategy.

Autophagy is induced by almost all conventional BC treatments and is considered a potential pharmacological target in the clinic. Autophagy inhibitors chloroquine (CQ) or hydroxychloroquine (HCQ) is in clinical trials for solid cancer therapy. One phase II trial suggested that the combined treatment of mTOR inhibitor everolimus and HCQ increased six-month median progression-free survival (PFS) in patients with BC 147. It has been suggested that anti-inflammatory polyphenolic stilbenoid resveratrol 148 induces autophagy, inhibits cancer stem cell proliferation, and suppresses the Wnt/beta-catenin pathway in BC cells 149. Resveratrol has also been shown to induce BECN1 independent autophagy-mediated cell death in MCF-7 cells, suggesting a novel mechanism of cell death in BC cells 150.

The phosphatidylinositol 3-kinase (PI3K)/AKT pathway, which is one of the significant survival pathways, is highly active in BC patients due to frequent mutation in the PI3K gene 151. Autophagy activators such as PI3K/mTOR inhibitors can also enhance cell cycle arrest and potential therapeutic strategies for treating metastatic BC patients 141. Everolimus (mTOR inhibitor) was the first rapamycin analog approved in combination with endocrine therapy for patients with hormone receptor (HR)-positive metastatic BC 151. Everolimus and aromatase inhibitor exemestane have significantly enhanced OS in HR-positive metastatic BC patients 152, 153. The Food and Drug Administration (FDA) has already approved these strategies for treating advanced metastatic BC patients. Other autophagy activators such as inhibitors of mammalian target of rapamycin complex (mTORC)-1 and mTORC2 provide promising results for treating metastatic breast tumors 151, 154, 155.

Cyclin-dependent kinase (CDK)-4/6 inhibitors interrupt cell division, and cell growth is the FDA approved drug for treating patients with metastatic BC. CDK4/6 inhibition also induces senescence and autophagy in BC cells, and blocking autophagy sensitizes BC cells to CDK4/6 inhibition, and enhances programmed cell death. These data suggest potential strategies to overcome the limitation of CDK4/6 inhibition in the clinic for metastatic BC patients 156, 157. The recent preclinical study also suggested that CDK4/6 inhibition in combination with mTOR inhibition effectively reduces metastatic triple-negative breast tumor growth in a mouse model 158, suggesting these novel strategies are essential for further investigation in the clinical setting. Metastatic colonization of the aggressive TNBC or HER2+ BC cells in the brain includes amplifying the epidermal growth factor receptor (EGFR) in the BC cells 159. Amplification of EGFR is often associated with loss of phosphatase and tensin homolog (PTEN), a positive regulator of downstream EGFR effectors, including PI3K/AKT pathways, which not only promotes cell survival, but also regulates c-Myc to facilitate metabolic reprogramming 160, 161. PTEN loss and elevated PI3K/AKT signaling were found in brain metastasis formation in BC patients 85, 87, suggesting that targeting autophagy and PI3K could be a potential strategy to treat BCBM patients.

## Conclusion

**Figure 1:**
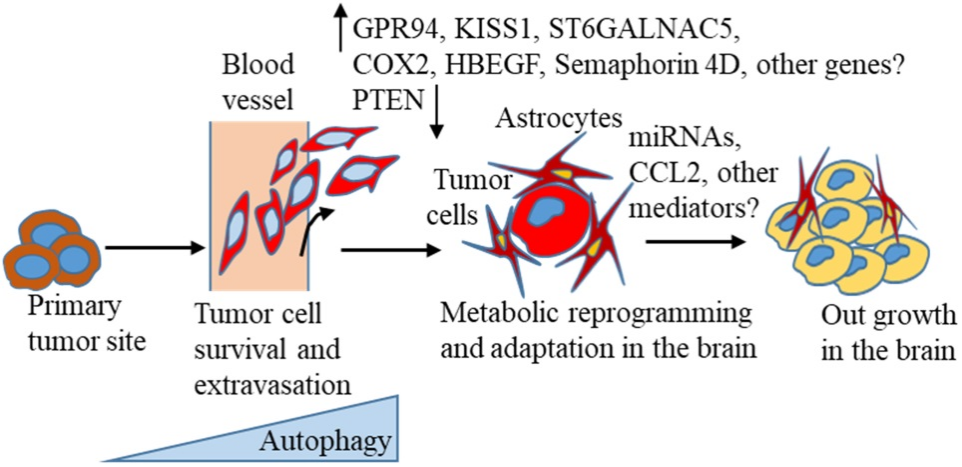
Autophagy-mediated cell survival under stress is involved in the invasiveness of BC cells. This contributes to cancer cell intravasation, survival in the circulation, escape from immune surveillance, and extravasation of cells 13, 14. Organotropic gene expression, gene signatures, and metabolic advantages are shown that mediate BC metastasis to the brain 82-84, 132, 136, 163. Autophagy-mediated metabolic adaptation, interaction with astrocytes, and mediator signaling 109, 132, 136, 138, 163, 164 are also proposed to modify cancer cells, which are able to establish and grow in the brain environment.

A large body of recent evidence suggests that autophagy is activated in tumor progression and different stages of the metastasis process 141, 162. Thus, targeting this pathway could be useful in treating BC distant metastasis, including BCBM. However, in reality, autophagy-targeting drugs have yielded contradictory or limited results, as summarised in this recent review 141, suggesting a precise understanding of the autophagic pathway related to BC progression and distant metastasis, including BCBM is extremely necessary. Despite the increasing incidence of brain metastasis, knowledge about the molecular mechanisms of brain metastasis development, and optimal treatment strategies is limited and less investigated than other metastasis formation. New markers for predicting BCBM occurrence in the primary tumors are urgently needed for early detection of the possibility of a high risk of brain lesion formation. To turn brain metastasis into a treatable disease, researchers need to understand the molecular changes that occur, as proposed in the context of autophagy (**Figure 1**), based on the current literature that suggests breast tumors escape from the primary site, evade immune surveillance, and finally, colonize successfully in the brain environment.

## Figures and Tables

**Table 1:** Pivotal genes/proteins participating in the process of autophagy and BCBM

Genes/Proteins	Autophagy in BC	BC	BCBM	Functions	References
Beclin 1	Lethal autophagy	Tumorigenesis	Not known	Heterozygous disruption of the beclin 1 is found in human BC, a tumor suppressor. It also enhances BC stem-like/progenitor cells.	69, 70
ATG4A	Survival autophagy	Tumorigenesis	Not known	ATG4A promotes BC stem-like phenotype.	59
ATG5	Lethal autophagy	Inhibition of tumorigenesis	Reduced BCBM	Downregulation of ATG5-dependent macroautophagy linked with BC growth and metastasis to the brain.	39, 71
ATG3, ATG7	Survival autophagy	Tumorigenesis	Not known	Promotes the survival of dormant BC cells and metastatic tumor recurrence.	58, 72
LC3	Survival autophagy	Tumorigenesis	Not known	TNBC cell proliferation, survival, migration, and invasion, and may contribute to tumor growth and progression.	73
LC3B	Survival autophagy	Tumorigenesis	Elevated in BCBM	Elevated levels are linked with shorter survival in TNBC patients.	68
P62/SQSTM1	Lethal autophagy	Tumorigenesis	Not known	Interacts with vimentin to enhance BC metastasis. Metastatic dormancy of BC.	72, 74
ULK1	Lethal autophagy	Inhibition of tumorigenesis	Not known	ULK1 phosphorylates Exo70 to suppress BC metastasis. Prognostic marker of BC patients.	75, 76
BIRC5	Lethal autophagy	Inhibition of tumorigenesis	Not known	Levels of BIRC5 and ATG7 were inversely correlated. A novel ATG12-ATG5 conjugate interactor. It induces lethal autophagy in BC cells.	77
MTA1	Survival autophagy	Tumorigenesis	Not known	A novel regulator of autophagy induces tamoxifen resistance in BC cells.	78
CEMIP and BiP	Survival autophagy	Tumorigenesis	Not known	Overexpressed, protective autophagy is observed in response to hypoxia, associated with tumor progression.	79
KISS1	Lethal autophagy	Inhibition of metastases development	Reduced expression of KISS1, enhances BCBM	Astrocytes promote BCBM via KISS1-mediated autophagy	80, 81
GRP94	Survival autophagy	Tumorigenesis	Induced BCBM	It is overexpressed in BCBM, involved in autophagy-mediated survival in endoplasmic reticulum stress.	82
FIP200/ATG17	Survival autophagy	Tumorigenesis	Protein expression higher in BCBM	A significant correlation is observed between higher FIP200/ATG17 expression in primary-BC tumors and shorter disease-free survival.	68
ST6GALNAC5	Not known	Tumorigenesis	Induced BCBM	It enhances BC cell adhesion to brain endothelial cells and their passage through the BBB.	83
Semaphorin 4D (SEMA4D)	Not known	Tumorigenesis	Induced BCBM	Semaphorin 4D/plexin B1 interactions mediate BCBM by promoting CTC transmigration through the BBB.	84
PTEN	Lethal autophagy	Inhibition of metastases development	Downregulated in BCBM	PTEN loss and elevated PI3K/AKT signaling are found in BCBM patients.	85

## Abbreviations

ATP: adenosine triphosphate
ATGs: autophagy-related genes
BC: breast cancer
BBB: blood-brain barrier
BCBM: breast cancer brain metastasis
BEN1: beclin-1
BCL2: B-cell lymphoma 2
BIRC5: baculoviral IAP repeat-containing protein 5
BiP: binding immunoglobulin protein
bFGF: basic fibroblast growth factor
CTC: circulating tumor cells
CCL2: C-C motif chemokine ligand 2
CXC: C-X-C motif chemokine ligand 8
CD: cluster of differentiation
CNS: central nervous system
CEMIP: cell migration-inducing protein
CQ: chloroquine
CDK: cyclin-dependent kinase
DMFS: distant metastasis free survival
EGFR: epidermal growth factor receptor
ET-1: endothelin-1
ECM: extracellular matrix
EIF4EBP1: eukaryotic translation initiation factor 4E binding protein 1
ERO1L: endoplasmic reticulum oxidoreductase 1 alpha
EVs: extracellular vehicles
FIP200: FAK family-interacting protein of 200 kDa
FOS: Fos proto-oncogene
FDA: Food and Drug Administration
GAPDH: glyceraldehyde 3-phosphate dehydrogenase
GRP94: glucose-regulated protein 94
GFAP: glial fibrillar acidic protein
GDNF: glial cell-derived neurotrophic factor
HR: hormone receptor
HER2: human epidermal growth factor receptor 2
HMGB1: high mobility group box 1
HCQ: hydroxychloroquine
IL-6: interleukin-6
IL-10: interleukin-10
IL-1β: interleukin-1β
IFN: interferon
ITPR1: inositol 1,4,5-trisphosphate receptor type 1
KISS1: kisspeptin 1
LC3A: light-chain 3A
LC3B: light-chain 3B
mTOR: mammalian target of rapamycin
MTA1: metastasis-associated 1
mTORC1: mammalian target of rapamycin complex 1
miRNAs: MicroRNAs
MIR345: MicroRNA 345
MMPs: matrix metalloproteinases
MIP-2: macrophage inflammatory protein 2
MMTV-PyMT: mouse mammary tumor virus-polyoma virus middle T antigen
NO: nitric oxide
NF-κB: nuclear factor kappa-light-chain-enhancer of activated B cells
OS: overall survival
PFS: progression-free survival
PERK: PKR-like endoplasmic reticulum kinase
PTEN: phosphatase and tensin homolog
PI3K: phosphatidylinositol 3-kinase
PCDH7: protocadherin 7
PTGS2: prostaglandin-endoperoxide synthase 2
ROS: reactive oxygen species
RFS: relapse free survival
RNA: ribonucleic acid
ST6GALNAC5: ST6 N-acetylgalactosaminide alpha-2,6-sialyltransferase 5
S1P: sphingosine-1-phosphate
SphK2: sphingosine kinase 2
S1PRs: S1P receptors
S1PR3: S1P receptor 3
STAT1: signal transducer and activator of transcription 1
SRS: stereotactic radiosurgery
TNBC: triple-negative breast cancer
TNFα: tumor necrosis factor-α
TGF: transforming growth factor
TME: tumor microenvironment
ULK1: Unc-51 like autophagy activating kinase 1
VEGFA: vascular endothelial growth factor A
WBRT: whole-brain radiation therapy
Wnt: wingless-type MMTV integration site family member
YAP: Yes-associated protein
